# Clinical decision support using machine learning and natriuretic peptides for the diagnosis of acute heart failure

**DOI:** 10.1093/ehjacc/zuae064

**Published:** 2024-05-16

**Authors:** Kuan Ken Lee, Dimitrios Doudesis, Johannes Mair, Nicholas L Mills, Study Group on Biomarkers of the ESC Association for Acute Cardiovascular Care, Bertil Lindahl, Jasper Boeddinghaus, Louise Cullen, Lori Daniels, Ola Hammarsten, Kurt Huber, Evangelos Giannitsis, Allan S Jaffe, Dorien M Kimenai, Konstantin Krychtiuk, Martin Möckel, Christian Mueller, Matthias Thielmann, Kristian Thygesen, Johannes Mair, Nicholas L Mills

**Affiliations:** BHF Centre for Cardiovascular Science, University of Edinburgh, 49 Little France Crescent, Edinburgh EH16 4SB, UK; BHF Centre for Cardiovascular Science, University of Edinburgh, 49 Little France Crescent, Edinburgh EH16 4SB, UK; Usher Institute, University of Edinburgh, Edinburgh, UK; Department of Internal Medicine III—Cardiology and Angiology, Innsbruck Medical University, Innsbruck, Austria; BHF Centre for Cardiovascular Science, University of Edinburgh, 49 Little France Crescent, Edinburgh EH16 4SB, UK; Usher Institute, University of Edinburgh, Edinburgh, UK

Acute heart failure accounts for 5% of all emergency medical admissions with the number of hospital attendances increasing rapidly in the last decade. Death in-hospital occurs in as many as 1 in 10 patients admitted with acute heart failure whilst nearly one in five patients do not survive 1 year following discharge from hospital.^1^ Previous studies have shown that delays in the diagnosis and provision of evidence-based therapies are associated with increased length of hospital stay and mortality.^2^ However, the diagnosis of acute heart failure may be challenging because other life-threatening conditions may present with similar symptoms and clinical signs. Furthermore, patients with heart failure frequently have multiple other cardiac and non-cardiac comorbidities that complicate the clinical presentation.

National and international guidelines recommend the use of natriuretic peptides to aid in the diagnosis of acute heart failure.^3^ B-type natriuretic peptide (BNP) is released as a prohormone in response to pathological conditions that increase volume and pressure within the heart. ProBNP is subsequently proteolysed into the biologically active BNP and an inactive N-terminal pro-B-type natriuretic peptide (NT-proBNP) fragment. Guidelines recommend thresholds of 300 and 100 ng/L for NT-proBNP and BNP, respectively, for the rule-out of acute heart failure, and the negative predictive value of these thresholds has been validated in multiple cohorts.^3^ However, the specificity and positive predictive value at these thresholds are low, and therefore age-specific rule-in thresholds of NT-proBNP (450, 900, and 1800 ng/L for those <50 years, 50–75 years, and >75 years, respectively) have been proposed.^4^

Despite guideline recommendations, natriuretic peptide testing has not been universally implemented, in part, due to concerns about clinical utility in a real-world setting. Previous studies investigating the diagnostic performance of natriuretic peptides have mainly been performed in relatively small, selected patient cohorts, limiting the generalizability of study findings across clinically important subgroups. Natriuretic peptide levels rise with declining renal function and increasing age. Conversely, patients with obesity have lower natriuretic peptide concentrations. Interpreting natriuretic peptide concentrations in individual patients can therefore be challenging particularly due to the increasing prevalence of comorbidities and obesity in this patient population. Indeed, in a recent individual-patient-level data meta-analysis, performance of guideline-recommended natriuretic peptide thresholds was heterogenous with a false negative rate between 1 in 10 and 1 in 5 in older patients and those with obesity or prior heart failure.^5^

To improve the utility of natriuretic peptides for the diagnosis of acute heart failure in the Emergency Department, individual-patient-level data were harmonized in 10 369 patients with suspected acute heart failure from 13 countries to train, test, and externally validate a clinical decision-support tool called CoDE-HF (Collaboration for the Diagnosis and Evaluation of Heart Failure).^5^ CoDE-HF used an eXtreme Gradient Boosting machine learning algorithm to combine NT-proBNP concentrations as a continuous measure and objective clinical variables that are known to be associated with acute heart failure to calculate the probability of acute heart failure for an individual patient (*Figure 1*). CoDE-HF had an excellent diagnostic performance overall and within all patient subgroups and was more accurate than any approach using NT-proBNP thresholds alone.^5^ Prospective studies are now underway to evaluate the impact of implementing this decision-support tool on healthcare resource utilization and patient outcomes. The application of machine learning has major potential to improve the utility of natriuretic peptides and other cardiac biomarkers,^6^ by supporting clinicians to make better and more individualized decisions in the diagnosis of acute heart failure.

**Figure 1:**
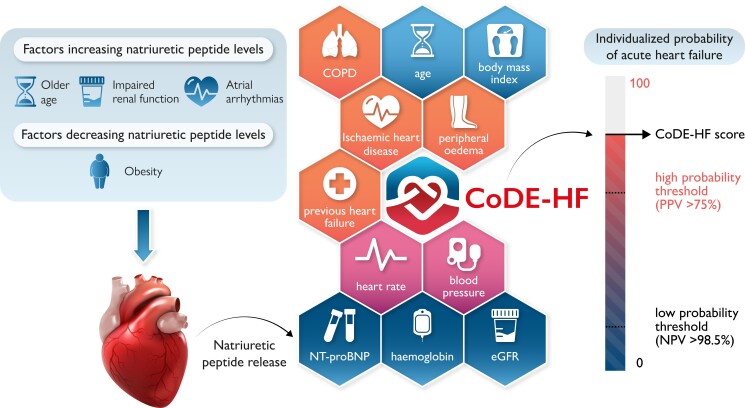
Factors influencing natriuretic peptide concentrations and the CoDE-HF algorithm. Multiple patient factors such as age, renal function, and body mass index can influence natriuretic peptide concentrations. A clinical decision-support tool called CoDE-HF (Collaboration for the Diagnosis and Evaluation of Heart Failure; https://decision-support.shinyapps.io/code-hf/) that combines NT-proBNP concentrations as a continuous measure and objective clinical variables (age, estimated glomerular filtration rate, haemoglobin, body mass index, heart rate, blood pressure, peripheral oedema, chronic obstructive pulmonary disease, and ischaemic heart disease) has been shown to be more accurate than any approach using NT-proBNP alone.

## Data Availability

The R code and anonymised data used to develop and validate the CoDE-HF score can be made available to researchers on request to the corresponding author.

## Appendix

Members of the Study Group on Biomarkers of the ESC Association for Acute Cardiovascular Care

Bertil Lindahl, MD^1^; Jasper Boeddinghaus, MD^2^; Louise Cullen, MD, PhD^3^; Lori Daniels, MD^4^; Ola Hammarsten, MD, PhD^5^; Kurt Huber, MD^6^; Evangelos Giannitsis, MD^7^; Allan S. Jaffe, MD^8^; Dorien M. Kimenai, PhD^9^; Konstantin Krychtiuk, MD^10^; Martin Möckel, MD^11^; Christian Mueller, MD^2^; Matthias Thielmann, MD^12^; Kristian Thygesen, MD^13^; Johannes Mair, MD^14^; Nicholas L. Mills, MD, PhD^9,15^

^1^Department of Medical Sciences, University of Uppsala, Uppsala, Sweden

^2^Cardiovascular Research Institute Basel (CRIB) and Department of Cardiology, University Hospital Basel, University of Basel, Switzerland

^3^Emergency and Trauma Centre, Royal Brisbane and Women’s Hospital, University of Queensland, Australia

^4^Department of Medicine, Sulpizio Cardiovascular Center, University of California, San Diego, La Jolla, CA, USA

^5^Department of Clinical Chemistry and Transfusion Medicine, University of Gothenburg, Gothenburg, Sweden

^6^Department of Medicine, Cardiology and Intensive Care Medicine, Wilhelminenhospital, and Sigmund Freud University, Medical School, Vienna, Austria

^7^Department of Cardiology, University Heidelberg, Germany

^8^Departments of Cardiology and Laboratory Medicine and Pathology, Mayo Clinic, Rochester, MN, USA

^9^BHF Centre for Cardiovascular Science, University of Edinburgh, Edinburgh, UK

^10^Department of Internal Medicine II, Division of Cardiology, Medical University of Vienna, Austria

^11^Division of Emergency Medicine, Charité-Universitätsmedizin Berlin, Germany

^12^Klinik für Thorax-und Kardiovaskuläre Chirurgie, Westdeutsches Herz- und Gefäßzentrum Essen, University of Duisburg-Essen, Germany

^13^Department of Cardiology, Aarhus University Hospital, Denmark

^14^Department of Internal Medicine III—Cardiology and Angiology, Innsbruck Medical University, Innsbruck, Austria

^15^Usher Institute, University of Edinburgh, Edinburgh, UK
